# Transcriptome Analysis and SSR/SNP Markers Information of the Blunt Snout Bream (*Megalobrama amblycephala*)

**DOI:** 10.1371/journal.pone.0042637

**Published:** 2012-08-06

**Authors:** Zexia Gao, Wei Luo, Hong Liu, Cong Zeng, Xiaolian Liu, Shaokui Yi, Weimin Wang

**Affiliations:** Key Lab of Freshwater Animal Breeding, College of Fisheries, Ministry of Agriculture, Key Lab of Agricultural Animal Genetics, Breeding and Reproduction of Ministry of Education, Huazhong Agricultural University, Wuhan, People's Republic of China; Centre of Marine Sciences & University of Algarve, Portugal

## Abstract

**Background:**

Blunt snout bream (*Megalobrama amblycephala*) is an herbivorous freshwater fish species native to China and has been recognized as a main aquaculture species in the Chinese freshwater polyculture system with high economic value. Right now, only limited EST resources were available for *M. amblycephala*. Recent advances in large-scale RNA sequencing provide a fast, cost-effective, and reliable approach to generate large expression datasets for functional genomic analysis, which is especially suitable for non-model species with un-sequenced genomes.

**Methodology and Principal Findings:**

Using 454 pyrosequencing, a total of 1,409,706 high quality reads (total length 577 Mbp) were generated from the normalized cDNA of pooled *M. amblycephala* individuals. These sequences were assembled into 26,802 contigs and 73,675 singletons. After BLAST searches against the NCBI non-redundant (NR) and UniProt databases with an arbitrary expectation value of E^−10^, over 40,000 unigenes were functionally annotated and classified using the FunCat functional annotation scheme. A comparative genomics approach revealed a substantial proportion of genes expressed in *M. amblycephala* tanscriptome to be shared across the genomes of zebrafish, medaka, tetraodon, fugu, stickleback, human, mouse, and chicken, and identified a substantial number of potentially novel *M. amblycephala* genes. A total number of 4,952 SSRs were found and 116 polymorphic loci have been characterized. A significant number of SNPs (25,697) and indels (23,287) were identified based on specific filter criteria in the *M. amblycephala*.

**Conclusions:**

This study is the first comprehensive transcriptome analysis for a fish species belonging to the genus *Megalobrama*. These large EST resources are expected to be valuable for the development of molecular markers, construction of gene-based linkage map, and large-scale expression analysis of *M. amblycephala*, as well as comparative genome analysis for the genus *Megalobrama* fish species. The identified SSR and SNP markers will greatly benefit its breeding program and whole genome association studies.

## Introduction

Blunt snout bream (*Megalobrama amblycephala* Yih, 1955), commonly known as Wuchang fish, is an endemic species in China. Its natural distribution is restricted to the middle and lower reaches of the Yangtze River, such as Liangzi (LZ), Poyang (PY) and Yuni (YN) Lakes [1], [2]. *M. amblycephala* is a species of ray-finned fish in the genus *Megalobrama*, which includes other three species *M. skolkoii*, *M. hoffmanni* and *M. pellegrini* [3]. Among these four species, *M. amblycephala* has been widely favored for its delicacy and recognized as a main aquaculture species in the freshwater polyculture system since 1960s in China [4]. Due to its successful artificial propagation and high economic value, *M. amblycephala* aquaculture industry has greatly developed in the past decades and its total output had reached 541,115 tons in 2001 [5]. As a consequence of fast domestication and over-fishing, however, germplasm resources of this bream are under threat of recession and mixture due to its artificial breeding [6], [7]. Nowadays, the cultured population of *M. amblycephala* is gradually exhibiting early sexual maturity, low growth rate and disease susceptibility. Therefore, applying genomics tools in the selection of elite broodstock has the potential to enhance the productivity and value of commercial production for this species.

Genetic marker discovery is the first step in the application of genomics to improve broodstock as these markers can be used for the creation of linkage maps and subsequent quantitative trait loci (QTLs) identification. Marker assisted selection (MAS) can be then employed by selecting broodstock based on genotypes at QTL that are relevant to economically important traits such as rapid growth, disease resistance and the control of early maturation. Currently, a limited collection of genetic markers is available for *M. amblycephala*, including mitochondrial DNA (mtDNA) [8], restriction fragment length polymorphisms (RFLP) and microsatellites [9], [10]. Many of the studies using genetic markers to analyze population structure in *M. amblycephala* have employed these genetic markers [2], [8], [11]. So far, only 60 microsatellites have been successfully developed for this species. Single Nucleotide Polymorphisms (SNPs) are highly abundant markers, which are evenly distributed throughout the genome and can be functionally relevant. Because of their potential for high genotyping efficiency, automation, data quality, genome-wide coverage and analytical simplicity [12], SNPs have rapidly become the marker of choice for many applications in genetics and genomics. They are suitable markers for fine mapping of genes and candidate gene association studies aimed at identifying alleles potentially affecting important traits. For instance, simultaneous analyses of thousands of SNPs have enabled genome-wide association studies for complex traits in chicken [13], pig [14], [15], cattle [16]–[18], horse [19] and sheep [20], [21]. However, such studies have not been possible in most aquaculture species because large numbers of SNPs have not been available. Developing a large set of SNP markers for genome analyses in *M. amblycephala* will facilitate fine mapping of QTL, improve the identification and exploitation of genes affecting important traits and enable selective breeding through genomic selection.

Although genome sequencing technology has become progressively more efficient over the past decade, the sequencing of complex genomes remains expensive. Expressed Sequence Tag (EST) sequencing provides an attractive alternative to whole-genome sequencing because this technique produces sequences of the transcribed portions of genes at a fraction of the cost of sequencing complete chromosomes. However, obtaining an EST database by traditional approaches was time-consuming and expensive and consequently restricted to a few model organisms [22], [23]. With the advent of the second-generation sequencing technologies [24], genetic/genomic resources for non-model species have become far more accessible and transcriptome sequencing is becoming one of the most important applications of second-generation sequencing in the field of ecology, evolution and genetics [25], [26]. Among the high throughput sequencing technologies, 454 pyrosequencing [27] allows for rapid transcriptome sequencing at a reasonable cost to any species. It is of particular interest in ecology and evolution [28]–[30] primarily because it yields longer sequencing reads than any other method (up to 600 bp), which allows more accurate de novo sequence assemblies that are often required for non-model organisms. Recently, 454 pyrosequencing was applied for the identification of gene-derived SNPs in a number of species such as *Eucalyptus grandis* [26], butterfly [31], lake sturgeon [32], coral [33], and pine tree [34].

The development of a number of genome resources facilitates both structural and functional analysis of the genome; however, only 20 EST sequences are available for *M. amblycephala* so far. In the present study, we used 454 pyrosequencing technology to perform an EST-based genome scan by sequencing cDNA pools of one-year-old *M. amblycephala* individuals in order to provide the most comprehensive gene sequence resources for *M. amblycephala* hitherto and to gather a large number of genetic markers (microsatellites and SNPs), which would greatly benefit its breeding program and whole genome association studies.

## Results and Discussion

### 454 pyrosequencing and assembly

**Figure 1:**
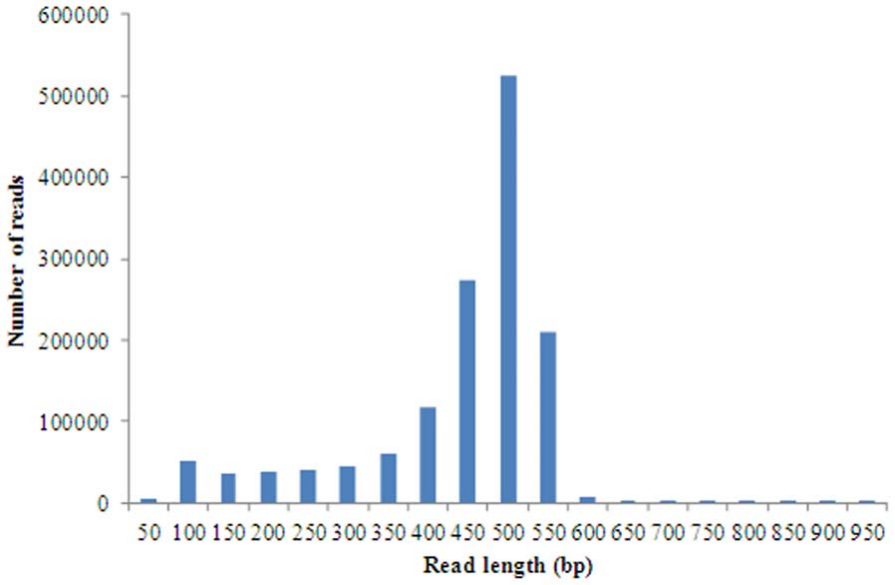
Frequency distribution of 454 sequencing read lengths for *M. amblycephala*.

We created a normalized cDNA pool using RNA extracted from different tissues of multiple *M. amblycephala* individuals. Pyrosequencing of this cDNA pool on a 454 GS XLR70 Titanium platform produced a total of 1,409,706 reads, with an average sequence length of 411 base pairs (SD = 117, range = 40–906; Figure 1), approximately 577 Mbp of sequence data. Assuming a similar number of genes and a similar average coding sequence length of 2,000 bp in *M. amblycephala* as in *Danio rerio* (37,855), average transcriptome coverage was estimated at 7.6×. To our knowledge, this is the first comprehensive study on the transcriptome of *M. amblycephala*.

**Figure 2:**
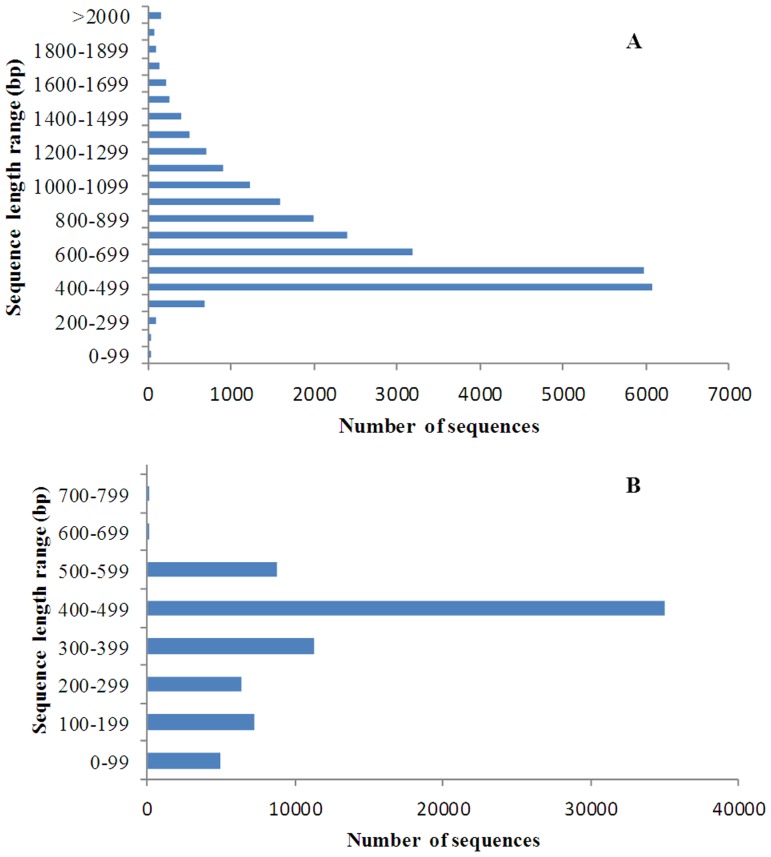
Overview of *M. amblycephala* transcriptome sequence length distributions for all contigs and singletons. (A) Sequence length distribution for all contigs; (B) Sequence length distribution for all singletons.

Clustering of the sequences resulted in 26,802 contigs, with an average length of 730 bp (SD = 331 bp). The contigs were consisted of 1,168,029 reads (83%) and the size distribution for these contigs is shown in Figure 2A. The assembly produced a substantial number of large contigs: 19,866 contigs (74.1%) were >500 bp in length, of which 4,685 (17.5%) were >1 kb. The majority of assembled reads were incorporated into the top 14,393 largest contigs, which included 74% of the obtained reads and accounted for 47% of the total assembled length. In addition, the assembly resulted in 73,675 singletons (mean length = 374±137 bp, Figure 2B). Singletons represent reads with similarities to other reads, but minor differences resulting in the exclusion from the clusters. The *M. amblycephala* 454 unigenes (contigs and singletons) sequences were uploaded to the website http://wbgd.hzau.edu.cn/additional/. A summary of the 454 sequencing data for *M. amblycephala* was presented in Table 1.

**Table 1:** Summary of 454 sequencing, assembly and analysis.

	Sequences (n)
Raw sequencing reads	1,409,706
Reads for assembly	1,280,970
Reads assembled	1,168,029
Contigs	26,802
Singletons	73,675
Total unique sequences	100,477

As compared with other recent studies [31], [33], [34]–[38], our results indicate that short reads from 454 sequencing runs can be effectively assembled and used to readily characterize the gene space of non-model organisms. The large number of reads in good length generated from a full plate pyrosequencing run on a 454 GS XLR70 Titanium instrument resulted in a high expected average transcriptome coverage depth (7.6×) for *M. amblycephala*. As expected with the increased read lengths from the 454 GS XLR70 Titanium instrument, the contigs in our present study were on average larger (mean = 730 bp) than those produced in previous studies using earlier 454 technologies with shorter reads (e.g., 197 bp [31]; 440 bp [33]; 500 bp [34]; 422 bp [35]; 300 bp [36]; 521 bp [37]; 547 bp [38]). In addition, a large percentage of our contigs were greater than 500 bp in length and high quality of assembled contigs was indicated by a high proportion of contigs matching to known proteins using BLAST searches.

### Gene identification and annotation

**Figure 3:**
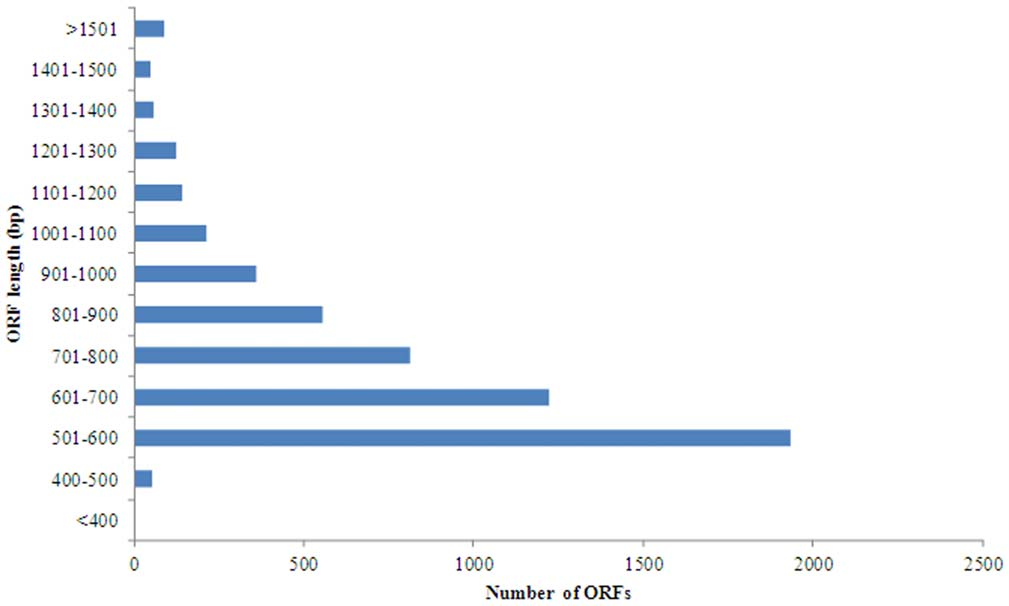
Open reading frame (ORF) length distribution from all contigs of *M. amblycephala*.

Putative gene identification was conducted by either EMBOSS software for identification of open reading frames (ORFs) or by BLASTx similarity search of public protein databases. Of the 26,802 *M. amblycephala* assembled contigs, 5,615 (21%) had an ORF longer than 200 bp, with an average length of 736 bp (SD = 244 bp, minimum = 305 bp, maximum = 3,017 bp; Figure 3).

**Figure 4:**
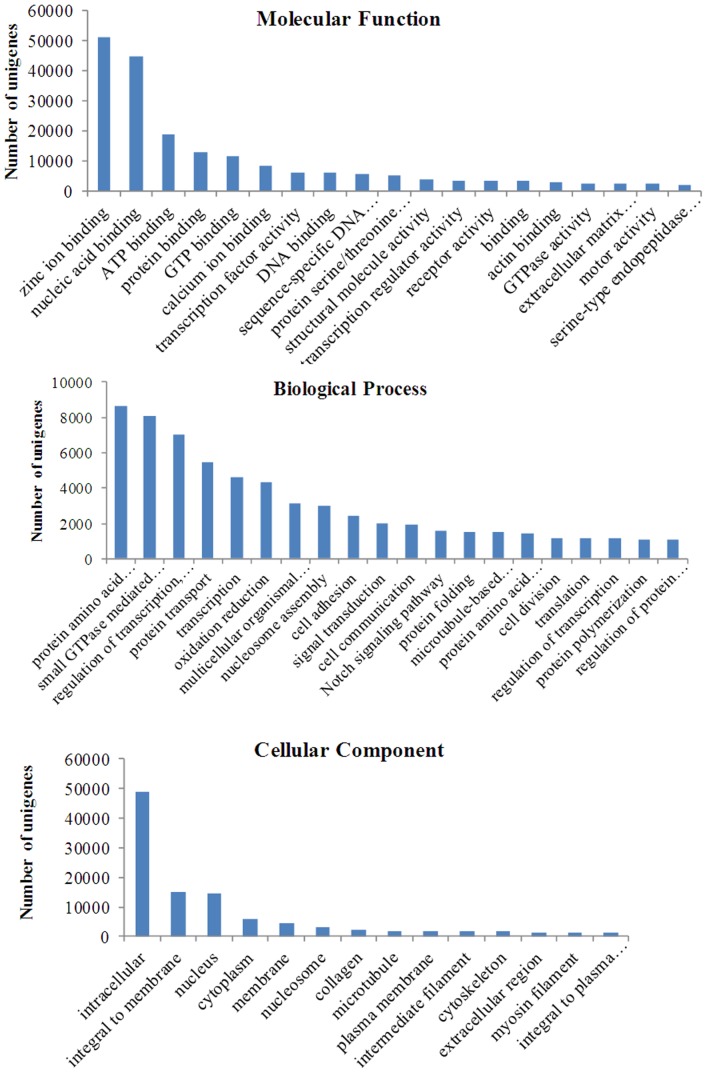
Gene ontology assignments for *M. amblycephala*. The annotated contigs and singletons from *M. amblycephala* 454 sequencing that matched various gene ontology (GO) categories.

BLAST searches for all contigs and singletons obtained from *M. amblycephala* were performed against the nr protein database using the BLASTx algorithm and an arbitrary expectation value of E^−10^ (Table S1). Among a total 100,477 unigenes (contigs and singletons), 40,687 (40.5%) showed at least one significant alignment to an existing gene model. The assembled transcripts were annotated against UniProt (Table S2). Of the 26,802 contigs, 17,378 (64.8%) returned an above cut-off Blast hits to the Uniprot database and the average length of these contigs was 803 bp. As for the 19,866 contigs with the length more than 500 bp, 13,915 (70.0%) showed significant hits to the Uniprot database. Of the 73,675 singletons, 24,629 sequences (33.4%) had Blast hits to the Uniprot database and the average read length of these singletons was 424 bp. Altogether, of the 100,477 unigenes, 42,007 (41.8%) had significant BLASTx hits to the Uniprot database and matched 20,674 unique protein accessions (Table 2). Gene ontology terms of biological processes, molecular functions and cellular components were showed in Figure 4. A large number of unigenes were assigned to a wide range of gene ontology categories.

**Table 2:** Summary of BLASTX search analysis of *M. amblycephala* unique sequences.

Dtabase	*M. amblycephala* hits*	Unique protein	% of total unique proteins
NR	40,687	19,230	
Uniprot	42,007	20,674	
Refseq/Ensembl			
Zebrafish	36,179	15,110	55% of 27,251
Medaka	28,740	12,302	50% of 24,661
Tetraodon	28,471	12,053	52% of 23,118
Fugu	29,112	14,890	31% of 47,841
Stickleback	29,736	13,122	48% of 27,576
Human	27,858	11,765	36% of 32,883
Mouse	27,776	11,201	37% of 30,100
Chicken	25,938	10,084	45% of 22,194
Cumulative unique (E^−10^)†	37,289	26,349	

Note:

* Number of significant (E^−10^) alignments using all *M. amblycephala* unique sequences as queries to search the listed databases.

† Cumulative unique totals were derived from the sum of unique gene/protein identities across all listed species.

Estimating the number of genes and the level of transcript coverage represented in an EST collection is an important issue for transcriptome sequencing projects, but is difficult or impossible without a completely annotated reference genome sequence. We indirectly evaluated transcriptome coverage breadth by determining the number of unique genes detected in our sequence collection using BLAST. The large number of sequences that matched in BLAST searches to unique proteins indicates that our 454 sequencing reads identified a substantial portion of the genes in *M. amblycephala*. A large portion of the *M. amblycephala* unique sequences had best BLAST hits to fish proteins, with a smaller portion (437 genes) hitting to other taxonomic groups. A similarly large number of these were assigned to a wide range of gene ontology categories, indicating that a wide collection of transcripts is represented by our sequence data. Furthermore, many of the contigs and singletons without BLAST hits likely represent additional genes not represented in the annotated protein databases that we searched, or genes that lack BLAST matches due to short length.

### Comparative analysis

In order to assess the level to which the *M. amblycephala* transcriptome has been captured, the unique sequences (100,477) were also searched against the NCBI Refseq and Ensembl databases. A number of significant hits were identified within zebrafish, medaka, tetraodon, fugu, stickleback, human, mouse, and chicken reference protein databases (Table 2). After removal of the redundant protein hits, 10,084–15,110 unique reference proteins were identified within zebrafish, medaka, tetraodon, fugu, stickleback, human, mouse, and chicken databases respectively (Table 2). The *M. amblycephala* 454 unigenes BLAST versus these 8 species were deposited in the website http://wbgd.hzau.edu.cn/additional/. The unique *M. amblycephala* sequences had the most hits (55%) to the unique proteins of zebrafish. A total of 37,289 cumulative unique genes were identified from *M. amblycephala* based on BLASTx searches against the Refseq/Ensembl database and that matched 26,349 unique protein accessions (Table 2). Taken together, these numbers provide strong evidence that this project has captured a majority of the *M. amblycephala* transcriptome.

**Figure 5:**
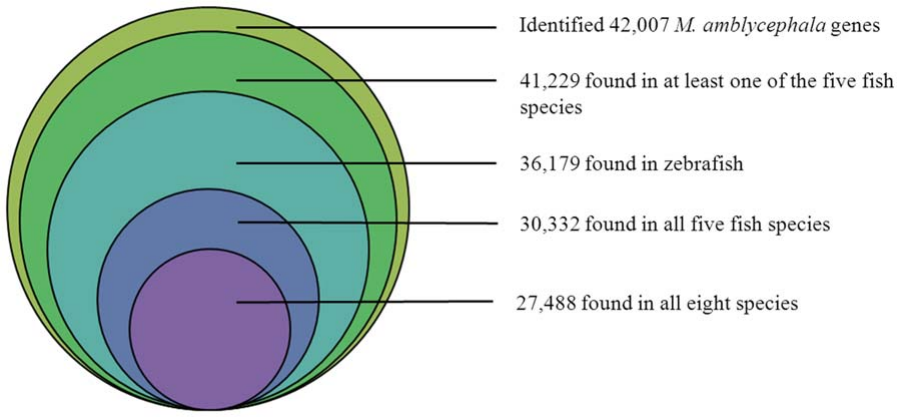
Conservation of *M. amblycephala* gene identities with other species. Number of *M. amblycephala* homologous genes identified from other species using BLASTX searches.

To assess the evolutionary conservation of the identified unique genes, the number of hits to unique genes in each species of zebrafish, medaka, Tetraodon, Fugu, stickleback, human, mouse, and chicken were compared. A total of 27,488 (65.4% of total number of unique *M. amblycephala* genes) putative known unique genes were found in all eight species; 30,332 (72.2%) were found in all five fish species; and 41,229 (98.1%) were found in at least one of the five fish species (Figure 5), indicating a high level of conservation of gene content among *M. amblycephala* and other teleost fish species.

### SSR/SNP markers identification

#### SSR markers

A total of 4,952 microsatellites were identified from 100,477 assembled unique sequences, including di-, tri-, tetra-, penta- and hexa-nucleotide repeats. Di-nucleotide repeats were the most common type in the EST dataset, with tri- and tetra-nucleotide repeats being present at much smaller frequencies. The characteristics of these microsatellites are summarized in Table 3. The total length of the unique sequences with microsatellites was 2.05 Mb and the average distribution distance of microsatellites was 9.53 Kb. After removing the microsatellites without enough flanking sequence for primer design, 3,372 microsatellites had sufficient flanking sequences (≥50 bp) on both sides to design primers for genotyping, which were distributed within 3,255 contigs. These microsatellites are expected to be useful for genetic linkage mapping and other genetic studies.

**Table 3:** Summary of microsatellite marker identification from *M. amblycephala* unique sequences.

Type	Number	Proportion in all SSRs	Frequency (%)^1^	Mean distance (kb)^2^	Microsatellites with 50 bp on both sides (Proportion)
Dinucleotide	3,107	62.74%	3.09%	15.18	2,025 (60.05%)
Trinucleotide	1,428	28.84%	1.42%	33.04	1,068 (31.67%)
Tetranucleotide	339	6.85%	0.33%	139.17	224 (6.64%)
Pentanucleotide	61	1.23%	0.06%	773.40	41 (1.22%)
Hexanucleotide	17	0.34%	0.02%	2775.13	14 (0.42%)
Total	4,952	100%	4.92%	9.53	3,372

Note:

1 Frequency = Total microsatellites number sought out/total number of unique sequences;

2 Mean distance = Total length of unique sequences/total SSR number.

In order to check the successful amplification proportion of these microsatellites, we randomly designed PCR primers with optimal expected product sizes for 160 microsatellites using Primer3. Of these 160 loci tested for successful PCR amplification in a single trial, 116 (72.5%) loci were successfully amplified in *M. amblycephala*. In addition, 71 (44.4%) of the tested microsatellites were polymorphic across panels of 40 individuals from the Liangzi Lake. The characteristics of these polymorphic microsatellites are listed in Table S3.

#### SNP markers

Considering the sequencing errors, a sequence variation was declared as a putative SNP or indel (insertion or deletion) when a mismatch was identified in contigs with four or more reads and the minor allele reads existed at least twice within contigs. A total of 56,109 putative SNPs were identified from assembled contigs (Table S4). These putative SNP types indicated an SNP rate of 1 per 302 bp of the transcribed sequences in *M. amblycephala*. A total of 72,020 insertions and deletions (indels) were discovered, that is, one indel per 158 bp of the transcribed sequences (Table S5).

**Figure 6:**
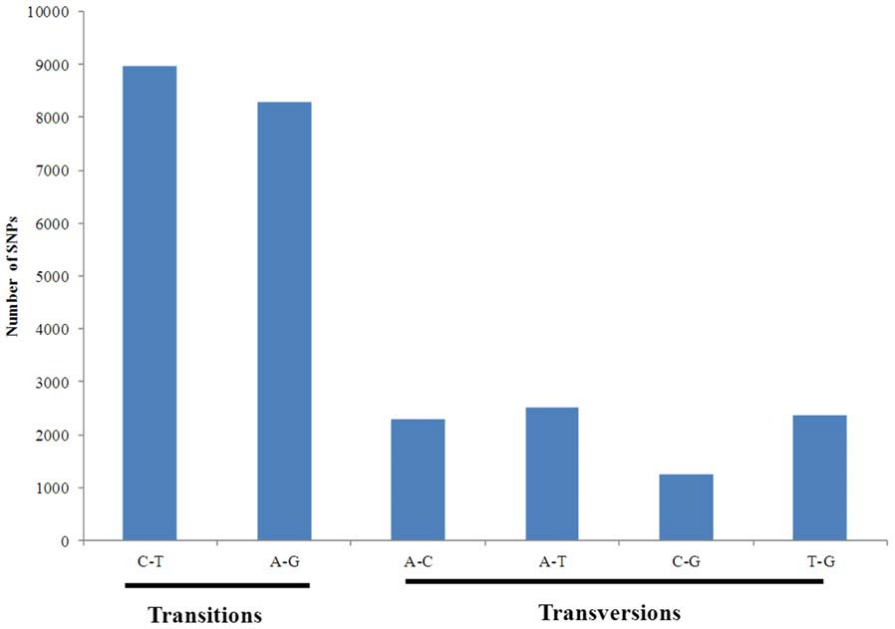
Classification of SNPs identified from 454 sequences of *M. amblycephala*.

**Figure 7:**
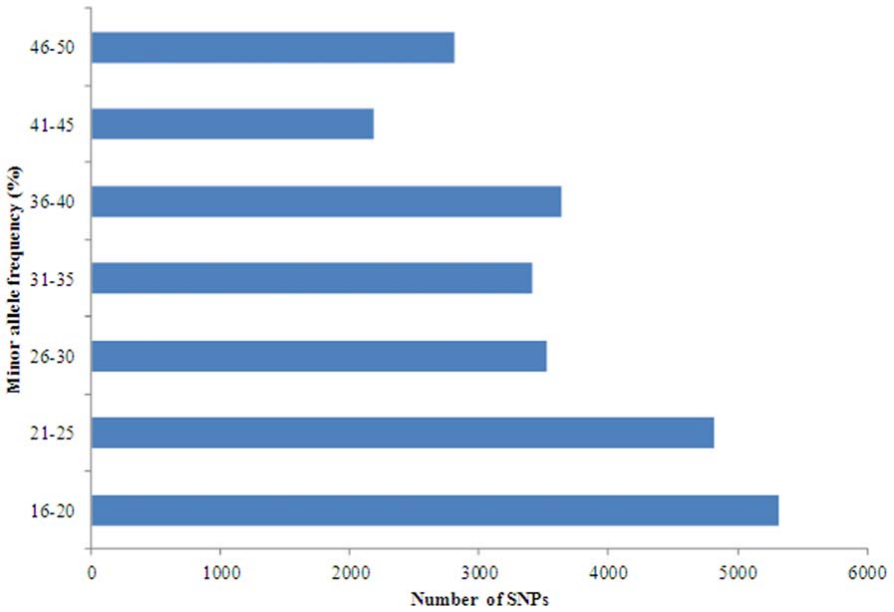
Distribution of minor allele frequencies of filtered SNPs identified for *M. amblycephala* from 454 sequences. The X-axis represents the number of SNPs with given minor allele frequency, while the Y-axis represents the SNP sequence derived minor allele frequency in percentage (>15%).

Putative SNPs/indels detected may be false positives, potentially arising from sequencing errors or misassembly of paralogous sequence variants or multisite sequence variants [39]. Therefore, in order to select SNPs/indels with high confidence, putative SNPs were screened based on several factors including surrounding sequence quality, absence of additional SNPs/indels in the flanking regions, and minor allele frequency. The setting of a minimum minor allele frequency >15% may help reduce false SNP/indel calling based on sequence errors. Additionally, multiple SNPs/indels located close to one another (<15 bp) often represent sequence errors and prevent the design of primers and probes for SNP/indels genotyping. Therefore, a requirement of no additional SNPs in the 15 bp flanking region around a putative SNP/indels was applied. After filtering, 25,697 SNPs were identified and these SNPs included 17,272 transitions and 8,425 transversions (Figure 6). The filtered SNP frequency in the transcribed sequences was one SNP per 401 bp of the transcribed sequences in *M. amblycephala*. A total of 23,287 filtered indels were identified with one indel per 392 bp of the transcribed sequences. Since the information on minor allele frequency is an important consideration in choosing which SNPs to be used in SNP arrays, the minor allele frequencies of SNPs in the discovery populations were estimated from the sequence data (Figure 7). The average minor allele frequencies were 30.9% in putative filtered SNPs identified for *M. amblycephala*.

The number of SNPs and their distribution in genes in a genome is important for the use of SNP in genetic analysis. A total of 12,314 *M. amblycephala* contigs were found to contain filtered SNPs, with the mean number of 2.09 SNPs per contig, while 11,241 contigs were found to contain putative indels with the mean number of 2.07 indels per contig. The number of unique Uniprot accessions hit by contigs containing SNPs was 8,489 in *M. amblycephala*, suggesting that putative filtered SNPs were identified from the vast majority of *M. amblycephala* genes.

Polymorphic genetic markers are important for research involving population genetic structuring, relatedness, the genetic basis of adaptive traits, and genetic selection [40], [41]. The second-generation transcriptome sequencing leads to superior resources for the development of such markers not only because of the enormous amount of sequence data in which markers can be identified, but also because discovered markers are gene-based. Such markers are advantageous on functional variation and the signature of selection in genomic scans or association genetic studies [42], [43]. Currently few genetic marker resources exist for *M. amblycephala*, wherein 454 pyrosequencing in the present study was shown to be an excellent method for large scale prediction of molecular markers for future genetic linkage and QTL analysis in non-model organisms this *M. amblycephala*. Given that these SSR and SNP markers were predicted fromtranscriptomic sequences, they are likely linked to protein-coding genes, and therefore might have substantial physiological implications [44].

Pooling of RNA samples from multiple individuals followed by transcriptome analysis using 454 sequencing provides an excellent opportunity to generate large numbers of SNP markers [23]. Previous studies have used 454 sequencing for finding large numbers of SNPs, which was performed using the whole genome sequence (WGS) as a reference for SNP determination [45]. However, developing such markers using 454 sequencing has also been proved being efficient in species without genomic resources. Beldade et al. [46] used over 20 outbred butterfly (*Bicyclus anynana*) individuals in their wing tissue cDNA library and were able to identify over 14,000 candidate SNPs. Parchman et al. [34] used normalized cDNA collections from multiple tissues and 4 individuals and detected 3,707 high quality SNPs with a minimum coverage depth of 8×. To infer SNP allele frequencies with a minimum reliability, it was recommended that a pool size of at least 10 individuals (20 chromosomes) is used [47]. Lower pool size would result in rather uninformative SNP frequencies estimates [47]. In our study, we used normalized cDNA collections from multiple tissues and 24 individuals with distinct growth performance to sequence, which resulted in a total of 34,771 filtered SNPs. Our results demonstrate that the number of pooling samples is an important factor for generating SNPs through the second-generation sequencing technologies.

### Conclusions

As a native fish species in China, *M. amblycephala* has become a main aquaculture species in the Chinese freshwater polyculture system. The genetic selection of this species is undergoing, which was funded by the earmarked fund for Modern Agroindustry Technology Research System entitled “Staple Freshwater Fishery Industry Technology System”. To date, the lack of genomics data available for this species has hampered the progress of its selective breeding. The 100,477 unique sequences in this 454 EST collection represent a major genomic level resource for *M. amblycephala*, and will be useful for comparative genomic studies in the genus *Megalobrama*. The EST contig library developed in this study can be used as a reference transcriptome for analysis of gene expression using cDNA microarray. Our results also demonstrated that the approach to sample individuals with diverse genetic backgrounds for reliable SNP identification allowed the ability to detect many SNPs across the entire genome. The SNPs identified in this study will provide a much-needed resource for genetic studies in *M. amblycephala* and will contribute to the development of high density, cost-effective genotyping platforms. Validation and testing of SNPs using high-density arrays will subsequently lead to the production of a SNP array, which would greatly benefit the breeding program and whole genome association studies for the performance and production traits in *M. amblycephala*.

## Materials and Methods

### Sample and RNA isolation

Twenty-four one-year-old *M. amblycephala* individuals from three natural populations were used for this study, of which 12 individuals have relatively highest growth rates (mean body weight 91.2±10.5 g, 4 individuals from LZ, PY and YN, respectively) and 12 individuals have relatively lowest growth rate (mean body weight 30.6±5.3 g, 4 individuals from LZ, PY and YN, respectively). Samples of 7 tissues including liver, kidney, muscle, testis, ovary, brain and heart were collected. The fish were euthanized with tricaine methanesulfonate (MS 222) at 100 mg/L before tissue collection. All the experimental procedures involving fish were approved by the institution animal care and use committee of Huazhong Agricultural University.

Total RNA was extracted from these samples using TRIzol® reagent (Invitrogen, Carlsbad, CA, USA) according to the manufacturer's protocol. RNA quality and quantity was measured using the NanoDrop 2000 (Thermo Scientific, Wilmington, DE, USA). All the samples were standardized to 200 ng/µL, and equal volumes of the same tissue samples from different individuals were combined into one pool, making a total of seven RNA pools. The pools were DNase-treated with Turbo DNA-free (Ambion) and purified with RNeasy Mini Kit (QIAGEN, USA) following the manufacturer's instructions; RNA quality and quantity was determined again at the end of the process.

### cDNA synthesis, normalization and 454 sequencing

The seven RNA pools were used for double-strand cDNA synthesis using SMART technology (BD Biosciences Clontech), and then the cDNA was purified using the QIAquick PCR Purification Kit (QIAGEN). Full-length cDNA sample containing equal amounts of cDNA from each library was normalized using the Trimmer kit (Evrogen, Moscow, Russia) according to the manufacturer's instructions. Normalization decreases the prevalence of highly abundant transcripts which should lead to an increase in the number of different transcripts represented in the sequence reads and consequently the number of SNPs available for analysis.

The normalized cDNA pool was sequenced in a single full plate run on the 454 GS-FLX Titanium series pyrosequencer (Roche Applied Science) according to the manufacturer's instructions at the SeqWright Incorporation (http://www.seqwright.com/). The 454 GS-FLX Titanium series reagents has the ability to sequence 400–600 million base pairs per run with 400–500 base pair read lengths.

### Sequence assembly

Prior to assembly, we used the 454 Newbler 2.3 software to perform adaptor, SMART primer and poly-A tail trimming and also quality filtering (threshold quality score = 20). The remaining reads were clustered using the MIRA V2.9.26x3 assembler [48] with the “de novo, normal, EST, 454” parameters, specifying a minimum read length of 40 nt, a minimum sequence overlap of 40 nt, and a minimum percentage overlap identity of 80%. Sequences after assembly comprised contigs, singletons and “debris” reads. Contigs based on at least two reads, singletons refer to single reads that clustered with other reads, but were eventually excluded from the final assembly. “Debris” reads may be of high-quality, but display no significant relationships to any other reads during the assembling process, representing very short reads, but also single mRNAs.

### ORF searching and gene annotation

All unique sequences obtained after the assembly were analyzed by EMBOSS 6.4.0.1 software [49] to search for ORFs. The putative protein sequences were also generated at the same time by EMBOSS, which could be used to analyze splice variation, determine paralogs, and assess gene families.

Putative gene names, protein domains, and gene ontology terms were assigned to all contigs and singletons (referred to as unigenes) that shared sequence similarity with previously identified genes annotated with those details. For gene name annotation, the unigenes were compared against Uniprot and nr protein sequence databases using BLASTx with a significance threshold of E<10^−10^. Gene names were assigned to each assembled sequence based on the best blast hit annotated with a gene name, as determined by parsing the GenBank record features ‘CDS’ and ‘Protein’ for that blast hit. Blast hits annotated with uninformative terms were encountered (e.g., ‘unknown’, ‘uncharacterized’, or ‘hypothetical’) were skipped in this analysis. For computational speed, the search was limited to the first 10 significant hits for each query.

To annotate assembled sequences with Gene Ontology (GO) terms describing biological processes [50], molecular functions, and cellular components, sequences were compared against the UniProt-TrEMBL database of protein sequences [51] using WU-blast. This database was chosen because it has been extensively annotated with GO terms [52]. Each query sequence was assigned GO terms based on those associated with the top blast match for that query, using custom Perl scripts to compare the blast results and the table of annotations. Because the Gene Ontology is hierarchical in structure, the GO terms annotated here can be traced back to one or more parent terms at a given level of detail. The NCBI Refseq protein and Ensemble databases (zebrafish, medaka, Tetraodon, Fugu, stickleback, human, mouse, and chicken) were also used to annotate the unique *M. amblycephala* genes.

### Microsatellite and SNP markers identification

All the unique sequences were used to search for microsatellite makers using Msatfinder [53] with a repeat threshold of six di-nucleotide repeats or five tri-, tetra-, penta-, or hexa-nucleotide repeats. Unique sequences containing 50-bp sequence on both sides of the microsatellite repeat were considered sufficient for primer design [54].

For putative SNP identification, reads were mapped onto the transcripts we assembled using SSAHA2 [55] with default parameter and then the SNPs were extracted using VarScan under default parameter. A sequence variation was declared as a putative SNP or indel (insertion or deletion) when a mismatch was identified in contigs with four or more sequences and the minor allele sequence existed at least twice within contigs.

In order to identify high quality SNPs, putative SNPs and indels identified as mentioned above were further screened following specific criteria based on the read depth, minor allele frequency, the quality of flanking regions and absence of other SNPs or indels within 15 bp flanking regions. Only those SNPs/indels with minor allele sequences representing more than 15% of the reads aligned at the polymorphic loci were declared as quality SNPs/indels. In addition, no extra SNPs/indels within 15 bp flanking regions were allowed.

### Data Deposition

The Roche 454 reads of *M. amblycephala* were submitted to NCBI Sequence Read Archive under the accession number of SRA045792.

## Supporting Information

Table S1 ***M. amblycephala*** **454 unigenes BLAST nr result.**
(RAR)

Table S2 ***M. amblycephala*** **454 unigenes UniProt result.**
(RAR)

Table S3 **The characteristics of the polymorphic microsatellites tested in a wild population of** ***M. amblycephala*** **.** The file contains microsatellites locus name, primer sequence, PCR annealing temperature, number of alleles, observed and expected heterozygosities and Hardy-Weinberg equilibrium analysis.
(DOC)

Table S4 **The putative and filtered SNPs for** ***M. amblycephala*** **.**
(RAR)

Table S5 **The putative and filtered indels for** ***M. amblycephala*** **.**
(RAR)
