# Supplementary material for: Transcriptome Analysis and SSR/SNP Markers Information of the Blunt Snout Bream (Megalobrama amblycephala)
Source: PLoS One. 2012 Aug 6;7(8):e42637. doi: 10.1371/journal.pone.0042637 (PMC3412804; doi:10.1371/journal.pone.0042637)
Supplement: Table S3 — The characteristics of the polymorphic microsatellites tested in a wild population of M. amblycephala . The file contains microsatellites locus name, primer sequence, PCR annealing temperature, number of alleles, observed and expected heterozygosities and Hardy-Weinberg equilibrium analysis. (DOC) [file pone.0042637.s003.doc]

Supplementary Table S1 The characteristics of the polymorphic microsatellites tested in a wild population of *M. amblycephala*

| Locus | Primer sequence (5’→3’) | Ta (°C) | Repeat Motif | Size range (bp) | *Na* | *Ho* | *He* | *P_HW_* |
| --- | --- | --- | --- | --- | --- | --- | --- | --- |
| EST1 | F: GCGATGATGGGAAAGATG  R: TGGAGGGCTAATTTGTTGA | 50.5 | (CA)_10_ | 181-203 | 3 | 0.28 | 0.36 | 0.09 |
| EST2 | F: AAAAAAAGAACGAGAGGG  R: TGATGATTTGGAGGAAGT | 50.5 | (GA)_15_ | 326-370 | 5 | 0.70 | 0.74 | 0.14 |
| EST3 | F: TGAACTGAGTAAAGTGAGGCTAAA  R TCGAACCGTGGAGGAGAT | 59 | (TCA)_10_ | 327-373 | 3 | 0.40 | 0.46 | 0.25 |
| EST4 | F: GCAGTGTTGGAGGTCGTG  R: CATACTGGAATGTTTGTTAGGA | 57.5 | (TG)_12_ | 135-151 | 5 | 0.70 | 0.72 | 0.09 |
| EST5 | F: TTTCTGCCACTGGAGACC  R: TTTGATGATGATTAGAGGAGG | 59 | (TG)_15_ | 281-351 | 6 | 0.69 | 0.72 | 0.00* |
| EST6 | F: TGTGTCAAAATGCGTTCA  R: TCTCCCCCCAAGCCTACC | 52 | (GT)_12_ | 217-325 | 7 | 0.74 | 0.81 | 0.00* |
| EST7 | F: GTTGAAAAGGGAGGGACT  R: TGGGGGACAAATAAAAGC | 56 | (GT)_10_ | 321-413 | 8 | 0.85 | 0.82 | 0.00* |
| EST8 | F: CCATCCAACACGCCGACTC  R: GCGGGAAATCACTTCATAACTCAA | 61.5 | (AC)_15_ | 290-380 | 6 | 0.83 | 0.67 | 0.00* |
| EST9 | F: GGGTTTGTCCATTACTGCC  R: TCCCTGGTCCGACTTTCC | 49.5 | (CAT)_11_ | 213-223 | 2 | 0.55 | 0.51 | 0.75 |
| EST11 | F: ATGCCAGTCTGCCAACAA  R: TTCAATGATCGTCCGTCTT | 58 | (TG)_11_ | 300-340 | 5 | 0.60 | 0.74 | 0.00* |
| EST12 | F: TCGTGCGAAGTAAACAAG  R: CAGGCAATAATAACAAAACC | 54 | (TCTT)_13_ | 205-259 | 17 | 0.78 | 0.86 | 0.27 |
| EST13 | F: TCTTTCACAAACAAACCCTT  R: GGATTATCAAACGCGGACT | 55.5 | (AC)_14_ | 221-251 | 5 | 0.73 | 0.79 | 0.14 |
| EST16 | F: GGGACTGCAAGAGGAGGA  R: GGATGGGATAGAGCAAGGA | 60 | (CA)_11_ | 174-186 | 4 | 0.63 | 0.70 | 0.17 |
| EST18 | F: CGAGTAAAATCCCAGAGG  R: ATATGCCATTTTCTCACTTC | 54.5 | (TC)_10_…(AC)_18_ | 206-258 | 6 | 0.80 | 0.80 | 0.00* |
| EST22 | F: TGCCTCGGTCTCACTCTG  R: AATCTCCTGGAACACTCTTTG | 59 | (AC)_11_ | 148-218 | 4 | 0.75 | 0.75 | 0.20 |
| EST23 | F: GCGATCATCAAGGCAACG  R: AGATTCATCAGCTCCTGTAGTGT | 57.5 | (AC)_12_ | 293-333 | 5 | 0.69 | 0.70 | 0.06 |
| EST24 | F: ACTGAAGCCCTCAACCTC  R: TCACAGCAGACATCCAACT | 58 | (GT)_10_ | 167-193 | 6 | 0.85 | 0.76 | 0.00* |
| EST26 | F: GTCAACATTCATACGGCG  R: TCATTTTTTAGGAGCGGG | 54 | (CA)_11_ | 222-272 | 5 | 0.73 | 0.77 | 0.12 |
| EST28 | F: CGACTCCTCGCTCACTTACA  R: ACGATCCCGTCCATCACT | 57 | (GT)_12_ | 179-253 | 7 | 0.78 | 0.71 | 0.00* |
| EST32 | F: TCAGCAGCTCCAGCACAG  R: ATCCACCATACCATCCAATCT | 57 | (TG)_10_ | 247-277 | 2 | 0.75 | 0.51 | 0.00* |
| EST33 | F: AAAATCCTCCCCAAAGTC  R: AGTGTTATCCTCAGCCACA | 54 | (AC)_16_ | 233-261 | 5 | 0.83 | 0.70 | 0.00* |
| EST34 | F: ACTTGCCACCAGGAGACC  R: TCCCAAAGAGCACCGACT | 58.5 | (GA)_11_ | 424-472 | 4 | 0.75 | 0.69 | 0.02 |
| EST37 | F: CACAAACCATAAACACAG  R: AATGCCCATAAAACACAC | 54 | (TG)_8_ | 170-188 | 6 | 0.78 | 0.76 | 0.00* |
| EST39 | F:ACTACAAACTACACGAGCACC  R:GCAGATTGTTGAGGGAGA | 55.5 | (TG)_17_ | 147-183 | 6 | 0.80 | 0.74 | 0.00* |
| EST43 | F:CGTAACCCAACTGTATCCG  R:GTTCACTCGTGCCCATCC | 58 | (CA)_14_ | 401-493 | 2 | 0.63 | 0.51 | 0.20 |
| EST46 | F:AGTATAAGTTGAGTGGGTG  R:TAAAGGGAAATTCTGGT | 50.5 | (ATCT)_25_ | 282-380 | 18 | 0.73 | 0.91 | 0.00* |
| EST47 | F:ACGGTGTCAGTTCAGCA  R:CTCCCACGACAGAAAGA | 50 | (AC)_19_ | 206-228 | 6 | 1.00 | 0.68 | 0.00* |
| EST51 | F:TGTTGATTGATGCTGCTC  R:CTCGACCCAAACGAAAGA | 51 | (TG)_13_ | 294-394 | 6 | 0.82 | 0.81 | 0.12 |
| EST53 | F:TGCGTTTCTGTTGATGGATG  R:CAGGTATGGTCGGAGTGGC | 56 | (TG)_16_ | 367-433 | 6 | 0.83 | 0.73 | 0.00* |
| EST57 | F:ACCCATAAACTCAAGACTACAT  R:TGATTTCAGACAGGCACA | 53.5 | (AC)_10_ | 314-386 | 3 | 0.58 | 0.46 | 0.13 |
| EST58 | F:TGGCAAATGAAGATGAAG  R:TTACAACGCACCACTGAC | 52 | (GATA)_12_ | 300-330 | 2 | 0.55 | 0.51 | 0.75 |
| EST61 | F:CAACGGAAACCAGACAGGA  R:CATCACAATGAGTTTGAGGCT | 52 | (CA)_13_ | 199-251 | 5 | 0.36 | 0.67 | 0.00* |
| EST64 | F:AATCCAGTCAGAGTCATC  R:AGTCGTTTGTGCAAGTAA | 55 | (CA)_12_ | 110-170 | 7 | 0.90 | 0.76 | 0.00* |
| EST66 | F:TCAATCAGGCATAAACAT R:AACTAACTAGCACGCAAA | 50 | (CA)_12_ | 270-330 | 5 | 0.85 | 0.79 | 0.00* |
| EST70 | F:AATGAACGCAAAGAGCAA R:TTCTGTGAATCCTTCCCTC | 55 | (TC)_11_ | 311-341 | 4 | 0.83 | 0.60 | 0.00* |
| EST75 | F:GCAGACCCTTTCAGACAA R:ATCAGACCACTGCTTTCG | 52.1 | (CA)_12_ | 326-354 | 4 | 0.79 | 0.66 | 0.01 |
| EST79 | F:CAACGGAAACCAGACAGGA R:CATCACAATGAGTTTGAGGCT | 52 | (CA)_13_ | 300-380 | 5 | 0.78 | 0.67 | 0.05 |
| EST80 | F:TCAGCAACCGTTCACATA R:GCAGACCCTTTCAGACAA | 54.5 | (TG)_11_ | 240-290 | 5 | 0.80 | 0.66 | 0.00* |
| EST84 | F:ATGTATTGGGTTGAGGTT R:GAGCTATGGACTCCGTTAT | 53 | (TG)_14_ | 250-306 | 9 | 0.56 | 0.80 | 0.00* |
| EST811 | F:TGGAGTTAGTGTCCGCTTGT R:AGGATACGGGTGAGTTCG | 56 | (TG)_13_ | 320-366 | 7 | 0.70 | 0.68 | 0.11 |
| EST821 | F:AGACGGAACAAACCCAGAG R:TATTTGTGCCCGAGTGAA | 53 | (CA)_10_ | 230-258 | 7 | 0.83 | 0.68 | 0.06 |
| EST831 | F:TTCACTCGGGCACAAATAA R:GCACTGACGGCACGGATC | 54.5 | (AC)_12_ | 207-231 | 5 | 0.73 | 0.72 | 0.10 |
| EST841 | F:TCACAGACCAAATGGCAACA R:GGGCGAATTACAAGCTACA | 54.5 | (CT)_10_ | 481-493 | 6 | 0.80 | 0.66 | 0.00* |
| EST851 | F:ATTGGTCCAGTCTGTTGT R:TGTATCTTGCACGCTCTA | 54.5 | (AAGA)_14_ | 277-307 | 9 | 0.75 | 0.75 | 0.10 |
| EST90 | F:CTTACAGACTCCGACAGG R:ATCCACGACTTCCAGAAC | 57 | (AC)_12_ | 206-298 | 9 | 0.51 | 0.78 | 0.00* |
| EST91 | F:AAAGTGTTGAGGGGGGGA R:CAGGAAGTTGGAAGGCGG | 57 | (TG)_11_ | 270-296 | 4 | 0.82 | 0.62 | 0.01* |
| EST94 | F:GCAGATTCATCGCTCCTC R:CTCCAGCACTCGAACTTTAC | 57 | (GT)_12_ | 396-460 | 6 | 0.80 | 0.75 | 0.00* |
| EST95 | F: CACTCGCTGTGGTGGAAG  R: GAAGATGTGCTATCTGGGTCA | 59 | (AC)_14_ | 370-386 | 2 | 0.63 | 0.50 | 0.19 |
| EST98 | F:TCATGCTTGAAGCGTGTTGC R:CGCCTGCCATCCTAAGTGTT | 57.5 | (AC)_16_ | 326-388 | 5 | 0.85 | 0.75 | 0.00* |
| EST99 | F:TTTCACCAGTGAGGAACTC R:AGATTAGCAGGCGTTTCA | 57.5 | (TG)_13_ | 208-278 | 7 | 0.80 | 0.69 | 0.00* |
| EST100 | F:GCGTATGAACGTCAGAGC R:TGTTGGATTATTATGGGATG | 57 | (GT)_20_ | 208-256 | 9 | 0.78 | 0.76 | 0.00* |
| EST106 | F:CCTCGCCATCTACAAGTG R:CGTCCAAGCAGCAAAACA | 60 | (CA)_14_ | 376-428 | 6 | 0.78 | 0.76 | 0.00* |
| EST108 | F:TGAGTAGATGCCTCTGCCAATA R:CTGCGATGGAATGTCTGTTG | 51 | (AC)_10_ | 360-396 | 4 | 0.54 | 0.66 | 0.00* |
| EST110 | F:GCCTGACAGTCTTCTGC R:GCTATCCGATTATCATTTAC | 59 | (AC)_13_ | 196-234 | 5 | 0.78 | 0.75 | 0.00* |
| EST116 | F:CTATTTACAGTTTCATGCTTTCCTC  R:ATCCCGTCCGCCGCTTACT | 62 | (AC)_13_ | 157-271 | 8 | 0.83 | 0.78 | 0.00* |
| EST129 | F:GTAAACAGAACTACAGAGGGAG  R:CTAATACGGCACAAGGGT | 57 | (AC)_19_ | 327-365 | 4 | 0.80 | 0.70 | 0.02 |
| EST140 | F:GTCTGGCTTATCATAAAGAG  R:GAGGAGGTGAGAACTGGA | 54 | (AC)_17_ | 205-227 | 2 | 0.60 | 0.49 | 0.19 |
| EST144 | R:GCTATCCGATTATCATTTAC  F:GCCTGACAGTCTTCTGC | 59 | (AC)_13_ | 368-406 | 6 | 0.88 | 0.74 | 0.00* |
| EST147 | F:ACTGGATGACTTTAGTTAGGGTTA  R:CAGAAACGGCTTATCAGACC | 57 | (TG)_12_ | 212-258 | 5 | 0.85 | 0.68 | 0.01* |
| EST148 | F:CTTGAGGGTGAGTAAATGA  R:TGAGGAACAGTCCAACAC | 54 | (GT)_14_ | 219-231 | 3 | 0.65 | 0.52 | 0.09 |
| EST166 | F:GGTACTGTTTGTGCTGGGC  R:CTGCTCACTCAACTTATTGTAGGTC | 60 | (GT)_16_ | 117-151 | 6 | 0.50 | 0.68 | 0.02 |
| EST167 | F:AGCAAACAGTCTGCCAACA  R:GGCGGTCTAGTGCATTTACGT | 57 | (TG)_10_ | 396-429 | 2 | 0.78 | 0.51 | 0.00* |
| EST179 | F:ATTCATTATGGCGTGCTG  R:TTCTTGGCTGAGGGTATT | 54 | (CA)_10_ | 397-421 | 4 | 0.70 | 0.67 | 0.00* |
| EST184 | F:TGGGAGATAGGAGCAAGA  R:TGAATGGCTACAAGGTTTT | 54 | (GT)_10_ | 171-185 | 4 | 0.75 | 0.63 | 0.39 |
| EST195 | F:GTGTAACGGTTATGAACGAGTG  R:TGGGAAAGGGAAAGTGTATG | 57 | (CA)_10_ | 278-314 | 2 | 0.55 | 0.50 | 0.75 |
| EST196 | F:GAAGCAGGTGAACATCGTG  R:GGGTAGGTATAGTGTAGGGTGA | 58 | (AC)_10_ | 160-200 | 3 | 0.76 | 0.64 | 0.07 |
| EST197 | F:GACAGCCTCGATTTACTCATCC  R:GAGCGTTTCACAGCCTTGC | 60 | (GT)_11_ | 436-486 | 5 | 0.64 | 0.67 | 0.00* |
| EST198 | F:TTAGTGTCGCCCATTTGTG  R:ATGTGCCTTGGTTGCTTG | 57 | (AT)_10_ | 294-382 | 5 | 0.83 | 0.71 | 0.00* |
| EST202 | F:ACTACCTGCTAAAATCTTGCC  R:CGAATGGAGTGATTAGGACC | 55 | (GT)_10_ | 190-230 | 4 | 0.50 | 0.48 | 0.74 |
| EST204 | F:TGTCCCGTAAACAAACCT  R:CCTCTGATGCTGGAAACT | 53 | (CA)_10_ | 159-183 | 5 | 0.82 | 0.69 | 0.02 |
| EST208 | F:GCATCTAATGAATCGTTATG  R:GTTTTCTTGGCAGGTGTC | 53 | (AC)_12_ | 180-206 | 5 | 0.88 | 0.68 | 0.00* |
| mean value |  |  |  |  | 5.3944 | 0.72 | 0.68 |  |

F: forward primer; R: reverse primer; Ta: annealing temperature; *N*a: observed number of alleles per locus; *Ho*: observed heterozygosity; *He*: expected heterozygosity; *P*_HW_: probability value by Markov chain method for the Hardy-Weinberg equilibrium (HWE). * denoted significant departure from HWE after Bonferroni correction (*P*<0.05).
